# Prevention across the spectrum: findings from an RCT of three programs aimed at reducing risk factors for both eating disorders and obesity

**DOI:** 10.1186/2050-2974-1-S1-O36

**Published:** 2013-11-14

**Authors:** Simon Wilksch, Tracey Wade, Susan Paxton, Sue Byrne, S Bryn Austin

**Affiliations:** 1 School of Psychology, Flinders University, Australia; 2 School of Psychological Science, La Trobe University, Australia; 3 School of Psychology, University of Western Australia, Australia; 4 Boston Children's Hospital, Harvard Medical School & Harvard School of Public Health, USA

## Objective

To date the fields of eating disorder prevention and obesity prevention have remained largely separate from each other. Many reasons exist to try to prevent these two problems simultaneously with the most compelling being that there is an overlap in risk factors (e.g., dieting).

## Methods

Approximately N = 2,000 Grade 7 and Grade 8 girls and boys from schools in Victoria, South Australia and Western Australia participated. Classes in each school were randomly allocated to one of three 8-lesson programs: Media Smart; Life Smart; the HELPP Initiative (Helping, Encouraging, Listening and Protecting Peers) or to a control condition (usual school classes) with risk factor and weight status assessments at baseline; post-program; 6-month follow-up and 12-month follow-up. Media Smart and HELPP have both shown promise in previous eating disorder prevention trials while Life Smart was piloted and developed for this RCT. All three programs target one or more risk factors relevant to both eating disorders and obesity.

## Results

12-month follow-up data was being collected at the time of abstract submission. This will be the first presentation of findings from this RCT.

## Conclusions

Interpretations will be made regarding the comparative efficacy of each program as well as investigations by age group and gender.

This abstract was presented in the **Prevention** stream of the 2013 ANZAED Conference.

